# GPU-Accelerated Framework for Intracoronary Optical Coherence Tomography Imaging at the Push of a Button

**DOI:** 10.1371/journal.pone.0124192

**Published:** 2015-04-16

**Authors:** Myounghee Han, Kyunghun Kim, Sun-Joo Jang, Han Saem Cho, Brett E. Bouma, Wang-Yuhl Oh, Sukyoung Ryu

**Affiliations:** 1 Department of Computer Science, Korea Advanced Institute of Science and Technology, Daejeon, Republic of Korea; 2 Department of Mechanical Engineering, Korea Advanced Institute of Science and Technology, Daejeon, Republic of Korea; 3 Graduate School of Medical Science and Engineering, Korea Advanced Institute of Science and Technology, Daejeon, Republic of Korea; 4 Harvard Medical School and Massachusetts General Hospital, Wellman Center for Photomedicine, Boston, United States of America; German Cancer Research Center, GERMANY

## Abstract

Frequency domain optical coherence tomography (FD-OCT) has become one of the important clinical tools for intracoronary imaging to diagnose and monitor coronary artery disease, which has been one of the leading causes of death. To help more accurate diagnosis and monitoring of the disease, many researchers have recently worked on visualization of various coronary microscopic features including stent struts by constructing three-dimensional (3D) volumetric rendering from series of cross-sectional intracoronary FD-OCT images. In this paper, we present the first, to our knowledge, "push-of-a-button" graphics processing unit (GPU)-accelerated framework for intracoronary OCT imaging. Our framework visualizes 3D microstructures of the vessel wall with stent struts from raw binary OCT data acquired by the system digitizer as one seamless process. The framework reports the state-of-the-art performance; from raw OCT data, it takes 4.7 seconds to provide 3D visualization of a 5-cm-long coronary artery (of size 1600 samples x 1024 A-lines x 260 frames) with stent struts and detection of malapposition automatically at the single push of a button.

## Introduction

Acute coronary syndrome (ACS), most frequently caused by the rupture of an unstable coronary plaque, is the leading cause of death in the industrialized countries [1, 2]. One of the most widely-used and very effective techniques to treat coronary artery disease is percutaneous coronary intervention (PCI), which expands narrowed coronary arteries with stents to restore blood flow in coronary arteries. However, stent deployment has a potential risk of stent thrombosis due to its malapposition, which is more frequently found in patients with ACS than those with stable angina [3–5].

In order to check whether a stent placement procedure has completed successfully without significant stent malapposition, clinicians can utilize the frequency domain optical coherence tomography (FD-OCT) because its high-speed and high-resolution imaging capability enables accurate visualization of stent struts deployed [6–8]. FD-OCT has been especially beneficial for evaluating stent implantation in ACS [9]. Since the high speed FD-OCT allows imaging of a long coronary artery in a single pullback, it provides detailed information about stent struts and their relation with the vessel wall using 3D visualization technologies. For example, 3D OCT analysis techniques have shown high performance in evaluating coronary artery bifurcation lesions and their morphological changes after stent crossover [10–12].

However, the current 3D visualization technologies have limitations as several researchers have pointed out. V. Farooq *et al.* [13] described it as follows:

“Currently, the main limitation of this technology is the need for off-line creation of 3D reconstructions—prototypes of current generation ‘real time’ (i.e. available peri-procedurally at the ‘push-of-a-button’) remain experimental, work in progress, and are limited by relatively poor image quality/resolution.”

and Kubo *et al.* [14] also discussed the issues with 3D visualization as follows:

“Although 3D visualization provides a powerful technique of representing the OCT data, it is currently time-consuming. Ease of use of this new technology will bring it closer to becoming a practical imaging technique in cardiac catheterization laboratories.”

They hoped for a practically usable system that is both time efficient and easy to use like a “push-of-a-button” framework.

Recently, researchers have used graphics processing units (GPUs) to accelerate intracoronary OCT systems [15–21] but they have focused on accelerating specific modules rather than the entire systems. They have concentrated on accelerating the OCT image reconstruction module and some of them also optimized the 3D volume rendering module. Zhang and Kang [19] accelerated OCT image reconstruction using various GPU-based algorithms. Jian *et al.* [20, 21] accelerated OCT image reconstruction by optimizing data transfers and supported 3D rendering based on volume rendering algorithms from NVIDIA [22]. However, no existing research has reported GPU acceleration of the entire process of OCT systems from reconstructing images to 3D rendering.

In this paper, we present the first, to the best of our knowledge, GPU-accelerated “push-of-a-button” framework for intracoronary OCT, which reconstructs cross-sectional images of the vessel from raw OCT data, segments stent struts, detects malapposition, and generates 3D visualization in a fully automatic way with the state-of-the-art speed performance. Our framework supports various modules from reconstructing images to 3D rendering as one seamless process by developing clear interfaces between each module. To overcome well-known difficulties using both CPU and GPU effectively [23], we have analyzed each module to achieve the best performance depending on the algorithmic characteristics by selectively using CPU and GPU. While the recent research reports [24] that the performance gap between CPUs and GPUs is only 2.5x on average, our framework provides more than 9 times faster performance than using CPU; it automatically provides 3D volumetric visualization of a 5-cm-long coronary artery (of size 1600 samples × 1024 A-lines × 260 frames) with detection of stent malapposition from raw OCT data in less than 4.7 seconds.

## Push-of-a-Button Framework for Intracoronary OCT

Our push-of-a-button framework for intracoronary OCT comprises several modules: an intracoronary FD-OCT system, FD-OCT image reconstruction from raw data acquired by the OCT system, stent region selection, feature segmentation including segmentation of guide-wires and stent struts and detection of malapposed stents, and 3D visualization, as illustrated in S1 Fig. In this section, we describe each module of the framework in detail.

### Intracoronary FD-OCT System

**Fig 1:**
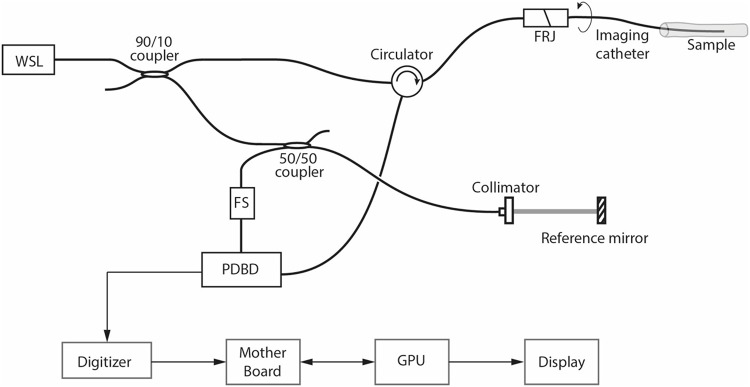
Schematic of the intracoronary FD-OCT system. WSL: Wavelength-Swept Laser, FRJ: Fiberoptic Rotary Junction, FS: Frequency Shifter, PDBD: Polarization Diverse Balanced Detection, GPU: Graphics Processing Unit.

The first module of our framework is an intracoronary FD-OCT system [25, 26], as depicted in Fig 1. A wavelength-swept laser (WSL) centered at 1,300 nm with wavelength tuning range and repetition rate of 120 nm and 110 kHz, respectively, was used as a light source. A frequency shifter (FS) in the reference arm removes the depth degeneracy enabling full-range imaging [27]. Both FD-OCT interference fringes corresponding to two orthogonal polarizations were detected separately by a pair of balanced receivers in polarization diverse balanced detection setup. Signals from each detector were sampled by a two-channel high-speed and high-resolution digitizer (Signatec PX14400, 14 bits, 2 channels) at 180 MS/sec. Each A-line consisted of 1600 sampled data. A pair of cross-sectional images corresponding to a pair of orthogonal polarization channels that consists of 1024 A-lines each was acquired at each longitudinal position of the vessel. Intracoronary imaging was achieved through an endoscopic imaging catheter. The axial and traverse resolutions were 9 *μ*m and 36 *μ*m, respectively. A high-precision and high-speed fiberoptic rotary junction (FRJ) spun the catheter providing 100 cross-sectional images every second.

After archiving the raw binary data acquired by the system for each polarization channel, we reconstructed FD-OCT cross-sectional images from the raw data as described in the next subsection.

### FD-OCT Image Reconstruction

The pseudocode of the FD-OCT image reconstruction algorithm is as follows:

for *n* *A-lines* of Raw Data:

# Background Subtraction

*Fringes* = *Fringes* − *background_fringe*

# Windowing Function Application

*Fringes* = *Fringes* * *windowing_function*

# FFT

*Depth* = FFT( *Fringes* )

# Demodulation and 2x Zero Padding

*Depth* = *Depth* [2/4] . zero . *Depth* [1/4]

# FFT Inversion

*Fringes* = inverse-FFT( *Depth* )

# Interpolation

*Fringes* = interpolation( *Fringes* )

# Dispersion Compensation

*Fringes* = *Fringes* * dispersion_compensation

# FFT for Image

*Depth* = FFT( *Fringes* )

# Power Spectrum with Log Scale

image = log(powerSpectrum( *Depth* ))

end

It comprises three fast fourier transforms (FFTs), demodulation and zero-padding, interpolation into uniform frequency space, dispersion compensation, and generation of intensity images in logarithmic scale. Because we implement full-range imaging by using a frequency shifter in the system [27], we perform FFT, demodulation for frequency shift (and 2x zero padding), and FFT back to time-domain to prepare the demodulated interference fringe data prior to applying actual OCT processing. For *n* A-lines of raw data, it performs a series of computations. First, it subtracts background signals from the A-line fringe signals to get the correct target image, and it adjusts the signals by multiplying window functions such as Hanning or Gaussian windows. The background data represents the default signals from the FD-OCT system without any sample signals, and the system generates the background data by blocking the sample signals. Prior to FFT, each A-line is zero-padded to have 2048 data. After applying FFT, it demodulates the frequency-shifted signals by aligning the zero depth to the zero electrical frequency. It then applies 2x zero padding to the demodulated signal and it performs the inverse FFT to obtain complex fringe signals. It performs interpolation to linear in *k*-space on the complex fringe signals by using the pre-calibrated mapping functions, and then the numerical phase-sensitive dispersion compensation [28]. Finally, by applying another FFT on the interpolated and dispersion compensated complex fringe signals, it creates an image by computing the log-scale intensity of the frequency signals.

As the pseudocode describes, the image reconstruction algorithm has high opportunities of parallel computations. First, the algorithm manipulates each A-line of raw data independent of information of other A-lines, which suits best for parallelization. Also, the operations on an A-line consist of simple arithmetic functions such as subtraction, multiplication, and FFT, which do not require high-performance computing features. We discuss how we utilize such features for acceleration using GPU in the next section.

### Stent Region Selection

Before segmenting various microscopic features related to stent struts, we automatically identify a subset of the input FD-OCT images that include stent struts. Unlike existing approaches, which set stent regions manually [29–31], we develop an algorithm to automatically detect the region as illustrated in S2 Fig.

**Fig 2:**
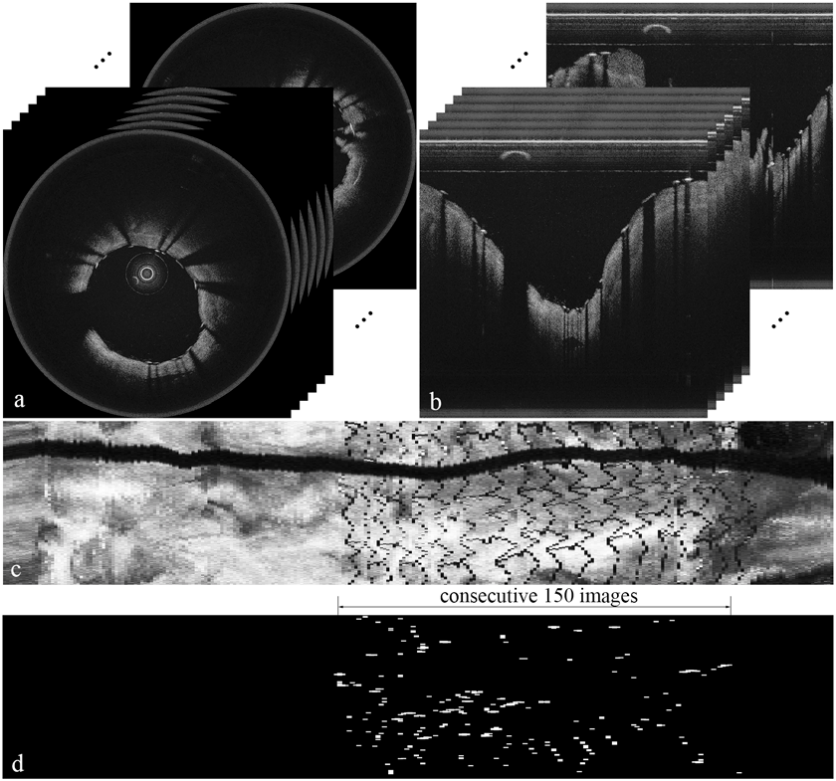
Cross-sectional images and their corresponding *en face* images. (a) FD-OCT images in polar coordinate. (b) FD-OCT images in Cartesian coordinate. (c) Sample *en face* image constructed from a series of FD-OCT images from a single pullback. (d) The *en face* image after applying the Laplacian filter to (c) and selecting only the pixel values greater than 240 from the resulting image, which shows consecutive 150 images.

First, we build an *en face* image from the entire cross-sectional images corresponding to a single pullback. From the cross-sectional images presented in polar coordinate (Fig 2(a)), we build an *en face* image as shown in Fig 2(c), where the height of the *en face* image is the number of A-lines in each cross-sectional image and the width is the number of all the cross-sectional images in a single pullback. We calculate the “pixel” value at the (i, j) coordinate in the *en face* image by computing the intensity average of all depth points at the j^*th*^ A-line of the i^*th*^ cross-sectional image.

Because the intensity values change substantially around stent struts, we apply the Laplacian filter, which detects edges and corners well, to the *en face* image and identify stent struts by selecting only the pixel values greater than a threshold 240. Fig 2(d) presents a resulting image after applying the Laplacian filter and selecting only the pixel values greater than 240. By using histogram equalization, which adjusts the image to make the brightest pixel value have the highest value, we can precisely detect stent struts and remove the other pixel values as noises.

Finally, we calculate an approximate number of cross-sectional images that include stent struts by dividing the length of a stent by the distance between images. For example, since the length of the stent in the exemplary image was 28 mm and the images are apart by 0.2 mm, we can estimate the maximum number of images with stents by 28/0.2, which amounts to 140 images. Thus, we calculate the numbers of all the pixel values greater than 240 in consecutive 150 images starting from the beginning of the cross-sectional images, and choose a sequence of maximum 150 images that has the largest number of the pixel values greater than 240 in them.

### Feature Segmentation / Malapposition Detection

The feature segmentation and malapposition detection module comprises several segmentation algorithms. Based on our previous work [32], we enhance various segmentation algorithms by removing manual adjustments and improving segmentation quality. S3 Fig illustrates the workflow of feature segmentation and malapposition detection. From the selected region of FD-OCT images, we segment the sheath of the catheter, a guide-wire, lumen, stent struts, and malapposed stents in order. We refer the interested readers to our earlier work [32] for the details of each segmentation algorithm, and we focus on the enhanced algorithms in this paper.

**Fig 3:**
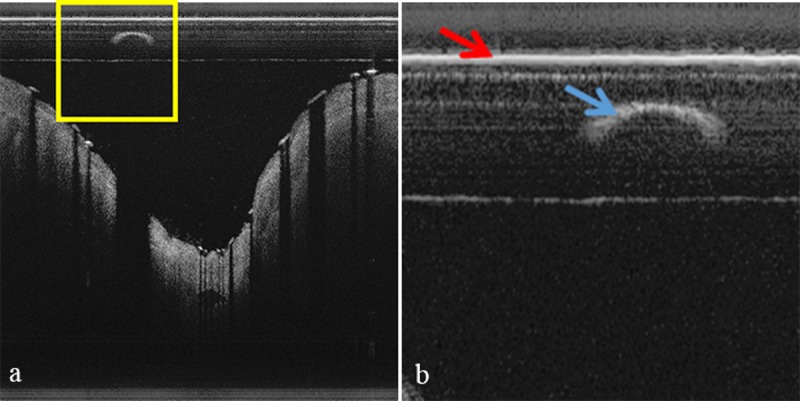
Catheter and guide-wire in a sample FD-OCT image. (b) is an enlarged image of the yellow box in (a). The left (red) arrow denotes the sheath of the catheter and the right (blue) arrow denotes guide-wire.

First, we automatically segment the sheath of the catheter by using its unique characteristic. Consider the yellow box in Fig 3(a), which is enlarged in Fig 3(b). FD-OCT images represent the sheath of the catheter as a horizontal line with strong signal as shown by the left (red) arrow in Fig 3(b). Using this observation, we automatically detect the sheath of the catheter by calculating the average horizontal intensity values in each image and selecting the area with the highest intensity.

**Fig 4:**
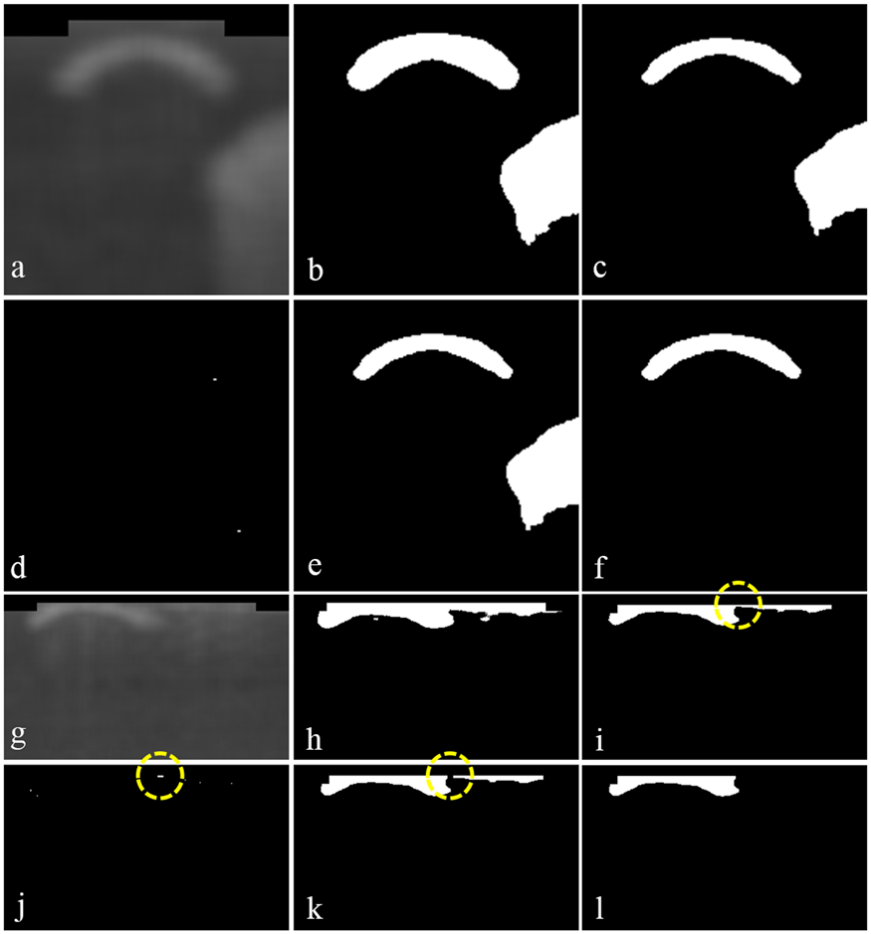
Method of precise guide-wire segmentation. (a)–(f) show a usual case where the morphological top-hat operation may not make any difference, whereas (g)–(l) exemplify such a case where the operation enhances the precision of guide-wire segmentation. (a) Set ROI from a blurred image. (b) Construct a binary image by Otsu’s method. (c) Apply the morphological erosion operation. (d) Apply the morphological top-hat operation. (e) Subtract the image in (d) from the image in (c). (f) Detect guide-wire by removing noises. (g) Set ROI, which is too wide. (h) Construct a binary image by Otsu’s method. (i) Apply the morphological erosion operation, whose resulting image includes connected guide-wire and noises denoted by a yellow circle. (j) Apply the morphological top-hat operation to detect the connected area denoted by a yellow circle. (k) Subtract the image in (j) from the image in (i) to disconnect guide-wire and noises as a yellow circle shows. (l) Detect guide-wire by removing noises.

Secondly, unlike our previous work, we now segment guide-wire automatically. In Fig 3(b), the right (blue) arrow denotes guide-wire. We detect the location of guide-wire in two steps: the first step identifies A-lines that include guide-wire and the second step detects the exact location of guide-wire. As in Ughi *et al.*’s approach [29], the first step approximately segments guide-wire; we construct an *en face* image like Fig 2(c) from a sample data, convert it to a binary image by applying Otsu’s method [33], and detect A-lines that include guide-wire by applying the morphological operations [34]. Note that performing only the first step cannot identify the precise location of guide-wire, and 3D visualization cannot use such imprecise guide-wire information. Fig 4 exemplifies how the second step segments guide-wire precisely. To show effects of the morphological top-hat operation, Fig 4(a)–4(f) show a usual case where the operation may not have any effect, whereas Fig 4(g)–4(l) show a case where the operation enhances the precision of guide-wire segmentation. Using the A-lines of guide-wire information obtained by the first step, we set region of interest (ROI) that may include guide-wire. Not to miss any guide-wire information, we set ROI conservatively: we add ± 40 pixels on both left and right sides of the A-line candidates. Fig 4(a) shows a sample image of setting ROI with extra zero padding at the top to avoid separation of guide-wire when we apply the morphological operation later. Then, we construct a binary image by Otsu’s method as in Fig 4(b), apply the morphological erosion operation as in Fig 4(c), and apply the morphological top-hat operation as in Fig 4(d). To detect guide-wire more precisely, we subtract the resulting image of morphological top-hat from the resulting image of morphological erosion as in Fig 4(e), which does not show much differences in this case. Finally, we segment guide-wire as in Fig 4(f) by detecting objects in images by connected component labelling (CCL) [35] and then removing noises. Note that the above algorithm may find a much wider ROI than the actual guide-wire information as Fig 4(g) illustrates. In such cases, applying Otsu’s method and then morphological erosion constructs one big object that includes both guide-wire and noises as in Fig 4(i). With the observation that the connected area between guide-wire and noises is narrow, we detect the connected area by using morphological top-hat as in Fig 4(j) and eliminate it to separate guide-wire from noises. Then, we can remove noises that are now separated from guide-wire.

**Fig 5:**
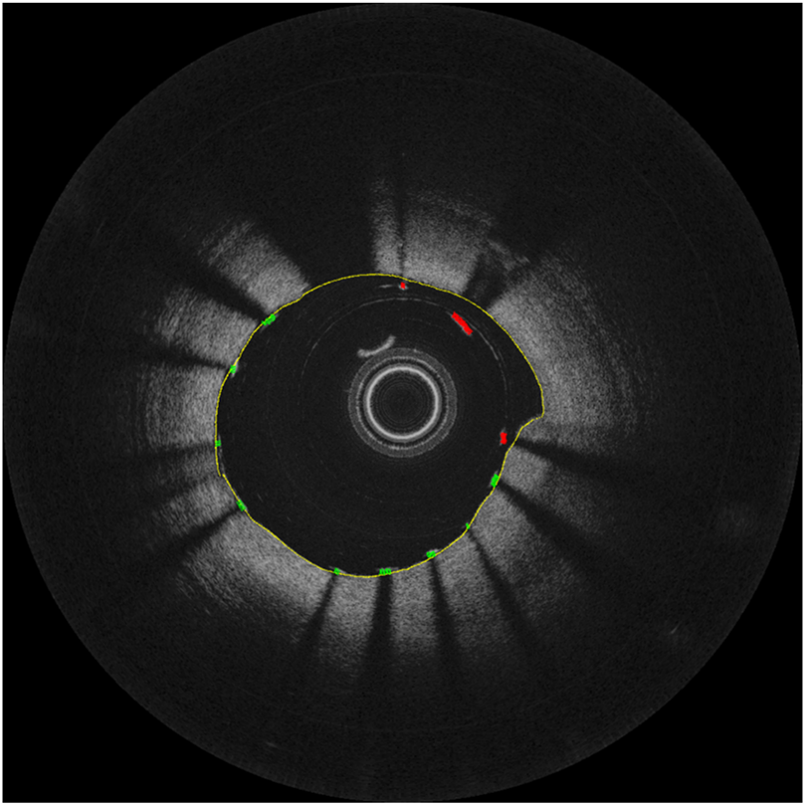
Sample image with the results of feature segmentation and malapposition detection. Lumen in yellow, apposed stents in green, and malapposed stents in red.

Then, we perform lumen segmentation and stent segmentation as we described in our previous work [32] to detect malapposed stents as shown in Fig 5. According to the consensus guideline [36], we can diagnose malapposition when the distance between the abluminal surface of the strut and the luminal surface of the artery wall exceeds the thickness of stent strut and polymer. In our patient data, we therefore consider more than 100 *μ*m as malapposition [37]. In our FD-OCT system, the distance between two vertically adjacent pixels is 6.5 *μ*m in polar coordinate OCT images. Therefore, we set malapposed distance as 15 pixels between the lumen and stent struts.

### 3D Visualization

The final module of our push-of-a-button framework is 3D visualization [25, 29, 31, 38]. Using the information collected by the feature segmentation and malapposition detection module, we obtain 3D visualization following the steps shown in S4 Fig.

**Cross-sectional Image Filtering and Adjustment** We preprocess FD-OCT cross-sectional images to make them suitable for high quality 3D volume rendering. First, we apply a smoothing filter to eliminate high frequency noises that happen typically in the cross-sectional images. Among various smoothing filters, we use a bilateral filter which preserves lumen edge information while removing high frequency noises well. Then, we adjust the intensity level of the images with histogram equalization. Because FD-OCT cross-sectional images without any adjustment tend to bias to dark intensities, we adjust the intensity of the images to distribute evenly from 0 to 255. After applying histogram equalization, the intensities of the remaining high frequency noises that are not removed by the bilateral filter become larger and they reveal themselves noticeable in 3D rendering. To eliminate such noises, we additionally adjust the intensity level: we set the intensity values lower than 128 to 0, and we readjust the intensity values greater than or equal to 128 to a range from 0 to 200 by interpolation.

**Feature Addition** Using the results of lumen segmentation, we eliminate the information inside lumen for rendering. For clear 3D volumetric visualization, we use intensity values between 0 and 200 for the vessel wall and intensity values between 201 and 255 for stent struts and guide wire.

**Polar to Cartesian Coordinate Transformation** Because intracoronary FD-OCT systems take images of the vessel wall with rotating catheters, the resulting FD-OCT images are in polar coordinate. For 3D visualization, we transform the images to Cartesian coordinate.

**Lumen Center Alignment** While transformation to Cartesian coordinate makes images in circular shapes, the centers of such images do not correspond to the lumen centers because the image centers depend on the location of catheter inside the vessel. To achieve more accurate and realistic 3D artery volume rendering, we calculate the lumen centers using the results of lumen segmentation, and we shift the lumen centers to the centers of images.

**3D Volume Rendering** Finally, we volume-render the FD-OCT images in Cartesian coordinate by implementing our own CUDA kernel functions. While the size of each image in Cartesian coordinate is 1024×1024, the total size of a resulting 3D rendering is too big to manipulate for closer observation. Thus, we resize the images to 512×512 so that we can rotate, cut, and expand the 3D volumetric image in real time. We also adjust the ratio of the widths and depths of the 3D image to visualize it more close to the real vessel. For realistic and clear visualization, we use different colors for the vessel wall, apposed and malapposed stent struts, and guide wire as we use different intensity values for them. We implement a ray tracing algorithm for 3D volumetric rendering in CUDA and we can observe the rendered 3D vessel in different directions by rotating them via an easy-to-use Qt [39] user interface.

## Methods for Acceleration by Selective GPU Processing

This section explains how we accelerate the performance of the entire framework by using both CPU and GPU selectively. We utilize various optimized implementations of image processing operations such as filtering and morphological operations provided by compute unified device architecture (CUDA) libraries from NVIDIA [40]. For each module in our framework, we selectively use CPU and GPU to achieve the best performance depending on the algorithmic characteristics of the module. Because the algorithms of FD-OCT image reconstruction, stent region selection, and 3D visualization mostly consist of simple operations on huge data, GPU programming alone performs the best, though it still requires careful configurations of CUDA programs for memory uses as we describe in the next subsection. However, feature segmentation and malapposition detection behave differently; their algorithms include various operations such as the ones that take less than 1 ms and the ones with complex computations. Therefore, well-designed uses of both CPU and GPU perform better than simply using only GPU. We discuss how we accelerate each module in following subsections.

### FD-OCT Image reconstruction

Because the FD-OCT image reconstruction algorithm composes simple operations on massive data, we achieve performance enhancement only by using GPU. We split the algorithm to multiple kernel functions around FFT calls as follows:

- Background subtraction & Window function application
- Demodulation for frequency shift & 2x zero padding
- Interpolation & Dispersion compensation
- Intensity image on a logarithmic scale

so that the functions on CPU can call the CUFFT library [41] without considering different characteristics of memory spaces.

To enhance the performance of our implementation by effectively using communications with CPU and various kinds of memory that GPU provides, we applied several techniques. First, we use page-locked or pinned memory transfers to take advantage of their highest bandwidth between the *host* on CPU and the *device* on GPU [42]. Because page-locked memory does not require page copies between the virtual memory and the system memory, the device does not need to communicate with the host to access the memory. Secondly, we parallelize memory copies from the host memory to the device memory and kernel execution using multi-streaming [20]. Multi-streaming enables memory copies and kernel execution to perform simultaneously, which almost hides the memory copy time via the kernel execution time. Finally, we use dual GPUs by evenly distributing CPU threads and data to process between two GPUs. Because intermixing CPU threads and GPUs degrades the performance, we enforce each GPU to communicate with only specific CPU threads.

### Stent Region Selection and 3D Visualization

Because the stent region selection module and the 3D visualization module consist of well-known operations and simple computations, we accelerate the modules utilizing well optimized OpenCV GPU library functions [43] such as image filtering, histogram equalization, and polar to Cartesian transformation. For submodules that the OpenCV library does not support such as *en face* image construction, noise removal, and feature addition, we accelerate them by our own CUDA kernel functions.

### Feature Segmentation / Malapposition Detection

Unlike the other modules that we could accelerate with simple CUDA programming, the feature segmentation and malapposition detection module is more challenging to accelerate because its submodules perform more complex computations. For example, the first step of stent segmentation detects whether each A-line in an FD-OCT image includes stent struts or not. In order to count the number of stent struts in each FD-OCT image, we perform clustering of the segmentation results constructed by the first step. The clustering submodule checks each A-line of an image to see whether its adjacent A-lines include stent struts in similar locations with the locations of its including stent struts. The computation for each A-line highly depends on its nearby A-line information, which is not suitable for CUDA programming. For such computations, running them on CPU performs better than on GPU.

Therefore, instead of using only GPU to accelerate the feature segmentation and malapposition detection module, we use both CPU and GPU selectively depending on the characteristics of submodules. The big question is how to divide the submodules to the ones to run on CPU and the others to run on GPU. We might measure the execution time of each submodule both on CPU and GPU, and execute each submodule on the processing unit that supports the best performance. However, the problem is not that simple; because the data transfer time between the host and the device is the major bottleneck in CUDA programming, we should not move data between the host and the device too frequently. Thus, we devise a mechanism to find a local optimal solution for the problem based on pre-measured execution time for each component of the modules.

Approximately speaking, we use GPU by default but if the execution time of a submodule on GPU is slower than the sum of the execution time on CPU and the data transfer time between the host and the device, we execute the submodule on CPU. To reduce the amount of unnecessary data transfers, we collect a sequence of consecutive submodules with same characteristics as one *group* to decide where to run them. Thus, we execute submodules that satisfy the following:

$$\begin{array}{c} T_{d}+T_{d^{\prime }}>T_{d2h}+T_{h}+T_{h2d}+T_{h^{\prime }} \end{array}$$ (1)

on CPU where *T*_*d*_ is the execution time of a group on GPU, *T*_*d*′_ is the time for auxiliary operations such as memory allocation on GPU, *T*_*d*2*h*_ is the data transfer time from the device to the host, *T*_*h*_ is the execution time on CPU, *T*_*h*2*d*_ is the data transfer time from the host to the device, and *T*_*h*′_ is the time for auxiliary operations on CPU, and execute the other submodules on GPU. To find the best combination of CPU and GPU uses, we implement every submodule in both C/C++ and CUDA that runs on CPU and GPU, respectively, and compare each implementation according to Eq (1). In the next section, we discuss our experimental results in detail.

## Results and Discussion

We report experimental results of our framework with pullback data acquired immediately after stent implantation, and we discuss how we achieve the push-of-a-button framework with the state-of-the-art performance. To show speed performance, we used averages of 30 execution results from when the host read raw binary data till when the framework displayed 3D visualization with one pullback data. To show accuracy of stent segmentation and malapposition detection, we evaluated segmentation results with 5 pullback data.

### Speed Performance

This section presents the speed performance of our framework. From a single pullback data that corresponds to 5.2 cm long coronary segment imaged with the longitudinal pitch of 200 *μ*m, our framework reconstructed 260 cross-sectional images. Then, it selected cross-sectional images with stent struts (130 cross-sections) automatically, performed feature segmentation and malapposition detection on the selected 130 images, and visualized them as a 3D volumetric image. Our software was built on Microsoft Visual Studio 2012, CUDA 5.0, Qt 5.0, and VTK 6.0, and our framework was built on an Intel(R) Xeon(R) CPU E5-2630 2.30 GHz, two Nvidia GPUs (GTX680), Samsung 32 GB RAM (DDR3 1333 MHz), and Microsoft Windows 8.

Table 1 summarizes the execution time of each module using CPU, CPU with 1 GPU selectively, and CPU with 2 GPUs selectively. Note that because we measured the entire execution time from pushing a button till displaying 3D images, the entire execution time is slightly bigger than the sum of the execution time of each module. While the recent research reports [24] that the performance gap between CPUs and GPUs is only 2.5x on average which is much closer than the myth that GPUs are orders of magnitude faster than CPUs, in our framework, selectively using 1 GPU and 2 GPUs in addition to CPU are more than 5 times and 9 times faster than using CPU, respectively. Table 1 also shows that selectively using CPU and 1 GPU performs better than using 1 GPU without using CPU for the feature segmentation and malapposition detection module.

**Table 1:** Execution time (in seconds) of each module in our framework using CPU, 1 GPU (selectively using CPU and 1 GPU), and Best (selectively using CPU and 2 GPUs).

Module		CPU	1 GPU	(1 GPU w/o CPU)	Best
FD-OCT Image Reconstruction	(260 frames)	29.640	0.844		0.458
Stent Region Selection	(260 frames)	0.788	0.397		0.397
Segmentation / Malapposition Detection	(130 frames)	9.736	6.358	(7.572)	3.254
3D Visualization	(130 frames)	5.280	0.828		0.627
Entire Execution Time*		45.445	8.429		4.731

* We measured the entire execution time from pushing a button till displaying 3D images, which is slightly bigger than the sum of the execution time of each module.

Table 2 compares the execution time of each module in our framework with the current state-of-the-art performance from the literature [19, 20, 29]. Because the existing techniques use 1 GPU, we compare the execution time of our framework using only 1 GPU with their execution time. For the FD-OCT image reconstruction module, we compared our result with Zhang and Kang’s [19] and Jian *et al.* [20]. Because Jian *et al.*’s algorithm is quite different from ours, we compared their algorithm with a simplified version of our algorithm that is similar to theirs. To the best of our knowledge, no existing research has reported speed performance of stent region selection. For the other modules, we compared our results with Ughi *et al.*’s [29], which used 150 images, except for 3D rendering because they did not report its execution time. While Jian *et al.* support 3D rendering [21] as well, they did not report the execution time of only 3D rendering. However, because our algorithm is also based on volume rendering algorithms from NVIDIA [22] as theirs, we believe that both algorithms have similar speed performance. Our framework shows the new state-of-the-art speed performance in every module. We describe how we achieve the performance enhancement by selectively using both CPU and GPU in this section. While we used two GPUs in our system, we ran each module on one GPU when we compare the speed performance between CPU and GPU to compare their performance differences precisely.

**Table 2:** Speed performance of each module in our framework and the current state-of-the-art techniques from the literature.

Module	Ours (1 GPU)*	Literature^†^
FD-OCT Image Reconstruction	809.5 KA-lines/second	672 KA-lines/second [19]
Simplified FD-OCT Image Reconstruction^‡^	2.27 MA-lines/second	2.24 MA-lines/second [20]
Stent Region Selection	0.397 seconds/260 frames	–
Segmentation/Malapposition Detection	6.358 seconds/130 frames	47.4 seconds/150 frames [29]
3D Visualization: Filtering ∼ Resizing	0.797 seconds/130 frames	24.0 seconds/150 frames [29]
3D Visualization: 3D Rendering	0.031 seconds/130 frames	–^§^

* Because the current state-of-the-art techniques from the literature use 1 GPU, we compare the execution time of our framework selectively using CPU and 1 GPU with their execution time.

^†^ Both our system and Jian *et al.*’s use GTX680 and Zhang and Kang’s [19] uses GTX590 that include 2 GTX580.

^‡^ In order to compare with Jian *et al.*’s algorithm [20], we simplified our algorithm to reconstruct FD-OCT images like what Jian *et al.*’s algorithm did, for example, like performing FFT only once.

^§^ While Jian *et al.* support 3D rendering [21] as well, they did not report the execution time of only 3D rendering. However, because our algorithm is also based on volume rendering algorithms from NVIDIA [22] as theirs, we believe that both algorithms have similar speed performance.

**FD-OCT Image Reconstruction** We split the FD-OCT image reconstruction algorithm into 4 kernel functions around 3 FFT functions and execute them only on GPU. S1 Table shows the execution time of each kernel and FFT function to reconstruct one FD-OCT image both on CPU and GPU. It shows that simple CUDA programming improves the performance immensely.

**Fig 6:**
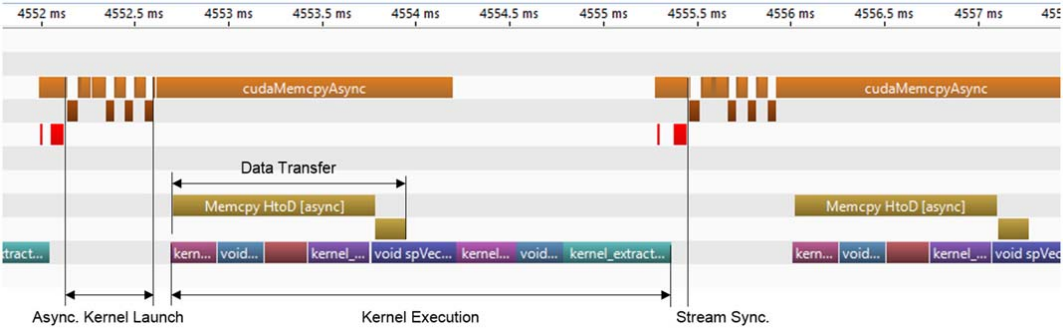
CUDA profiler timeline for FD-OCT image reconstruction.

We parallelize the data transfers between the host and the device and kernel executions [20]. Fig 6 illustrates that the data transfers from the host to the device are asynchronous (Memcpy HtoD [async]), which allows the kernel functions to execute concurrently with the data transfers. Using two GTX 680 GPUs, our algorithm processes 1.5 MA-lines in every second where we use 2048 A-lines (2 polarization channels×1024 A-lines) of raw binary data to reconstruct one FD-OCT image. Our algorithm is faster than the best GPU accelerated FD-OCT image reconstruction algorithms [19, 20]. In Zhang and Kang [19], the full-range 1024-pixel FD-OCT is the closest one to our FD-OCT system, and the LIFFT-D (linear spline interpolation with FFT and numerical dispersion compensation) algorithm is the closest one to our algorithm. Because we need both directions of data transfers, we should compare the 2-way limited speed by PCI-E. Then, their algorithm reports 672 KA-lines/second while ours reports 809.5 KA-lines/second. To compare with Jian *et al.*’s algorithm with 2.24 MA-lines/second [20], we revised our algorithm to execute similarly to theirs like performing FFT only once, and our revised algorithm reports 2.27 MA-lines/second.

**Stent Region Selection** We implemented both C/C++ code to run on CPU and CUDA code to run on GPU for stent region selection, and measured their execution time. As S2 Fig illustrates, stent region selection has 5 submodules: *en face* image construction, laplacian filtering, histogram equalization, noise removal, and region selection. S2 Table shows the execution time of each submodule on CPU and GPU omitting memory-related execution time such as memory allocation, deallocation, and data transfers between the host and the device. While some submodules execute faster on CPU than on GPU as gray rows in S2 Table show, we observed that the total execution time using GPU only is shorter than using both CPU and GPU because of data transfers between the host and the device.

**Feature Segmentation / Malapposition Detection** We implemented both C/C++ code and CUDA code for feature segmentation and malapposition detection. Because this is the most complex module in our framework, we implemented its 6 submodules consisting of 141 functions. S3 Table shows the execution time of 6 submodules on CPU and GPU. While the total execution time on GPU was about 1.39 times faster than on CPU, we observed that some functions execute faster on CPU than on GPU without much overhead for data transfers between the host and the device.

To achieve aggressive acceleration of feature segmentation and malapposition detection, we compared the execution time of the functions on CPU and GPU using Eq (1). We first identified functions and submodules that ran slower on GPU using the execution time of each function on CPU and GPU. To minimize unnecessary data transfers between the host and the device, we grouped consecutive functions and submodules together. S4 Table shows the experimental results of 13 groups that ran slower on GPU. According to Eq (1), we compared the execution time on GPU and the execution time on CPU with data transfer time. For example, the execution time of Group 2 on CPU with the data transfers is slower than the execution time on GPU. On the contrary, Group 7 shows that running it on CPU indeed performed better than on GPU. Among 13 groups, 10 groups executed faster on CPU. Using the experimental results, we used both CPU and GPU accordingly, which makes the execution time of feature segmentation and malapposition detection 1.53 times faster than on CPU as Table 1 shows.

**3D Visualization** S5 Table shows that when a problem suits well for parallelization, simple CUDA programming outperforms programs on CPU. While the Image Resizing submodule executes slightly faster on CPU, we use only GPU for 3D visualization because the performance gain was little and the actual execution time difference was negligible. Our 3D rendering algorithm is based on volume rendering algorithms from NVIDIA [22] as in Jian *et al.*’s [21].

### Accuracy

To show the accuracy of stent segmentation and malapposition detection in our framework, we evaluated segmentation results with 5 pullback data.

**Stent Region Selection** Because 1 of 5 pullback data showed that all of its cross-sectional images had stent struts, we analyzed the accuracy of stent region selection with the other 4 pullback data, and Table 3 shows the accuracy results. For 4 pullback data, the stent region selection algorithm missed 16 FD-OCT images and selected 26 more images compared to the ground truth. A simple stent region selection algorithm found the range of FD-OCT images that included stent struts effectively and reasonably precisely.

**Table 3:** Accuracy of stent region selection.

**Sequence ID**	**Ground Truth**	**Our Result**
1	106–235	105–234
2	139–238	139–248
3	70–219	85–234
4	87–236	87–236

**Fig 7:**
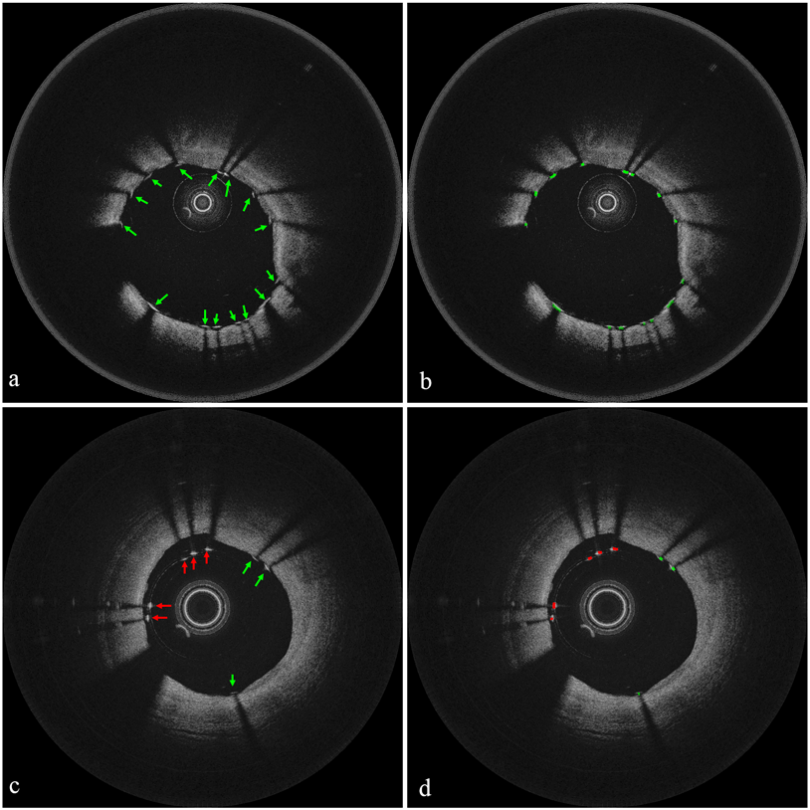
Results of stent segmentation and malapposition detection. (a) Result of manual stent segmentation. (b) Result of automatic stent segmentation. (c) Result of manual malapposition detection. (d) Result of automatic malapposition detection.

**Stent Segmentation and Malapposition Detection** Fig 7 shows the results of both manual and automatic stent segmentation and malapposition detection. Using the manual inspection results by two independent observers as the ground truth, we analyzed the accuracy of our automated stent segmentation and malapposition detection using 3 pullback data, which amounts to 360 FD-OCT images. One trained cardiologist manually inspected all the images and identified stent struts in them. To obtain the inter-observer reliability, another independent observer analyzed the same images for stent segmentation and malapposition detection. For the intra-observer reliability, each observer analyzed the same images twice. Table 4 shows some statistical measurement of the inter- and intra-observer reliability of our manual stent segmentation and malapposition detection. For stent strut assessment, we used Kendall’s rank correlation which is a nonparametric method used for ordinal variables. For malapposition detection, we calculated overall agreement and kappa value which is a chance-corrected indicator of agreement. The results showed that both measurements are in high correlation and excellent agreement.

**Table 4:** Inter- and intra-observer reliability.

	Inter-observer	Intra-observer
Kendall’s *τ*-b (95% CI)*	0.94 (0.92–0.95)	0.99 (0.99–1.00)
Overall Agreement^†^	98.6%	99.9%
Kappa Value (95% CI)^†^	0.78 (0.72–0.84)	0.98 (0.96–1.00)

* Results for stent segmentation.

^†^ Results for malapposition detection.

Table 5 shows the accuracy of automated stent segmentation and malapposition detection. To validate our stent segmentation and malapposition detection, we used 305 FD-OCT images by selecting every 2 frames in 5 pullback data. The sensitivity for stent segmentation was 88.3%. For malapposition detection, the sensitivity was 94.7% and the specificity was 94.7%. Note that we do not present the specificity for stent segmentation because strut identification results have no well-defined true negative [44].

**Table 5:** Accuracy of stent segmentation and malapposition detection.

	Stent Segmentation	Malapposition Detection
Number of Patients	5	5
Number of Frames	305	305
True Positive	2,473	142
False Positive	179	122
True Negative	–	2,201
False Negative	329	8
Sensitivity, % (95% CI)	88.3 (87.0–89.4)	94.7 (89.4–97.5)
Specificity, % (95% CI)	–	94.7 (93.7–95.6)

While our automatic stent segmentation and detection of malapposition have shown a promising diagnostic performance, further validation will be followed for detection of stent struts with very weak intensities or shadows.

### 3D Visualization

**Fig 8:**
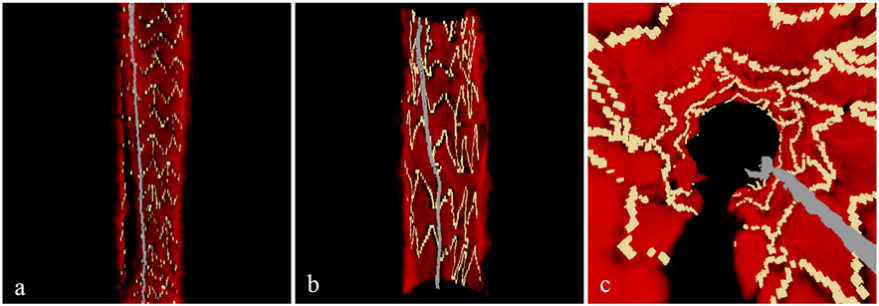
Cutaway views with segmented stents and guide-wire. Data acquired with (a) 200 *μ*m and (b) 100 *μ*m longitudinal pitches, respectively. (c) Fly through view (100 *μ*m pitch).

**Fig 9:**
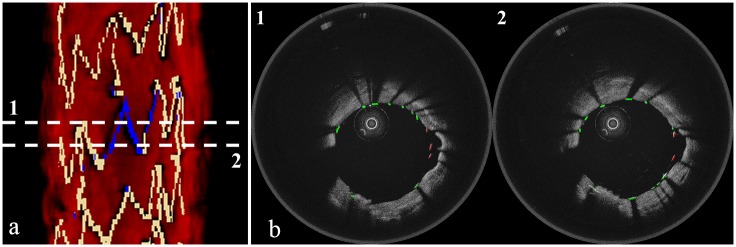
Cutaway view and its corresponding 2D images. (a) Cutaway view with properly apposed stents in yellow and malapposed stents in blue (100 *μ*m pitch). (b) Corresponding 2D images with apposed stents in green and malapposed stents in red.

Fig 8 shows sample images generated by the 3D visualization module in our framework. Fig 8(a) presents a cutaway view that illustrates the vessel wall in red, stent struts in yellow, and guide-wire in gray. Fig 8(b) shows a similar cutaway view for a different pullback, and Fig 8(c) shows its corresponding fly through view. While the cross-sectional images of the pullback in Fig 8(a) were apart by 200 *μ*m in longitudinal direction, the images of the pullback in Fig 8(b) and 8(c) were apart by 100 *μ*m. Fig 9(a) additionally shows malapposed stent struts in blue. Its corresponding 2D images in Fig 9(b) clearly show malapposed stent struts indicated by blue arrows.

## Conclusion

We present the very first “push-of-a-button” framework for intracoronary FD-OCT that reconstructs 3D volumetric images including stent struts from raw binary data with the state-of-the-art speed performance. Our framework acquires raw patient data using its FD-OCT system, reconstructs cross-sectional images, segments various features, and provides 3D visualization of the coronary artery systematically and automatically as a single coherent system. By selectively using both CPU and GPU based on empirical data, our framework reports the best performance for each of its modules compared to the current state-of-the-art techniques; it takes 4.7 seconds to provide 3D visualization of a 5-cm-long coronary artery. Our automatic stent segmentation and detection of malapposition have shown a promising diagnostic performance. Further validation in large patients data is warranted.

## Supporting Information

S1 Fig Workflow of the push-of-a-button framework for intracoronary FD-OCT. Boxes denote modules and shades denote data.
(TIF)

S2 Fig Workflow of stent region selection. Boxes denote submodules and shades denote data.
(TIF)

S3 Fig Workflow of feature segmentation / malapposition detection. Boxes denote submodules and shades denote data.
(TIF)

S4 Fig Workflow of 3D visualization. Boxes denote submodules and shades denote data.
(TIF)

S1 Table Execution time (in milliseconds/frame) of each function in FD-OCT image reconstruction on CPU and GPU. (DOCX)

S2 Table Execution time (in milliseconds/frame) of each function in stent region selection on CPU and GPU. (DOCX)

S3 Table Execution time (in milliseconds/frame) of each submodule in feature segmentation and malapposition detection on CPU and GPU. (DOCX)

S4 Table Execution time (in milliseconds/frame) of each group that may run faster on CPU in feature segmentation and malapposition detection. (DOCX)

S5 Table Execution time (in milliseconds/frame) of each submodule in 3D visualization on CPU and GPU. (DOCX)
