# Supplementary figures and images for: GPU-Accelerated Framework for Intracoronary Optical Coherence Tomography Imaging at the Push of a Button
Source: PLoS One. 2015 Apr 16;10(4):e0124192. doi: 10.1371/journal.pone.0124192 (PMC4400174; doi:10.1371/journal.pone.0124192)

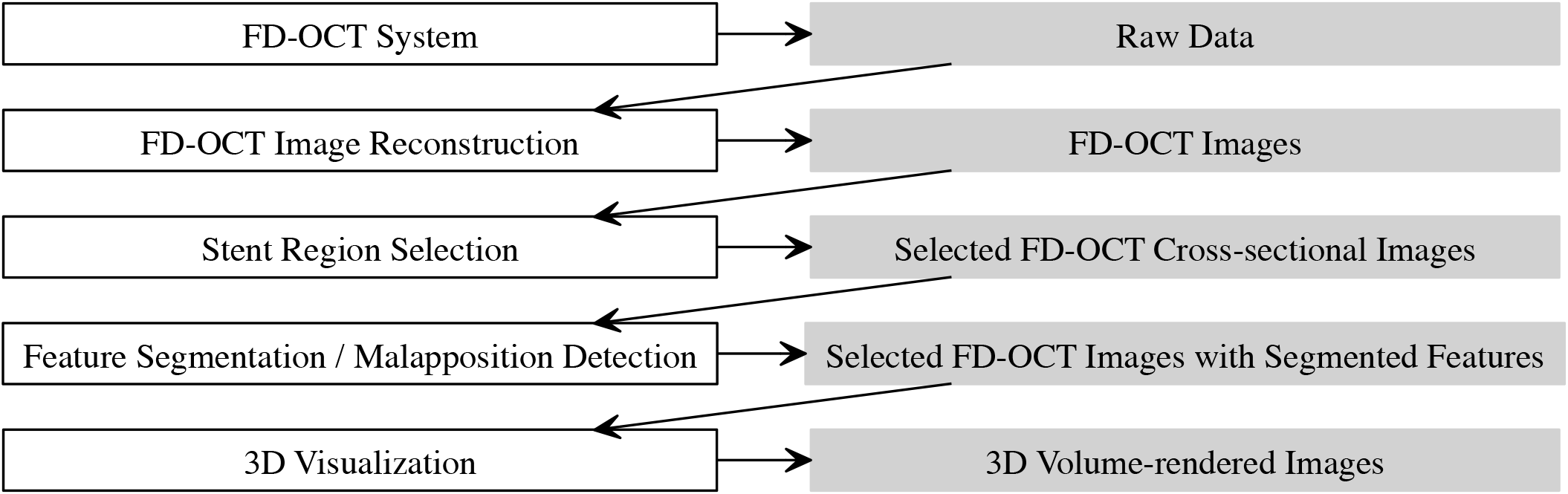

Supplement: S1 Fig — Boxes denote modules and shades denote data. (TIF) [file pone.0124192.s001.tif]

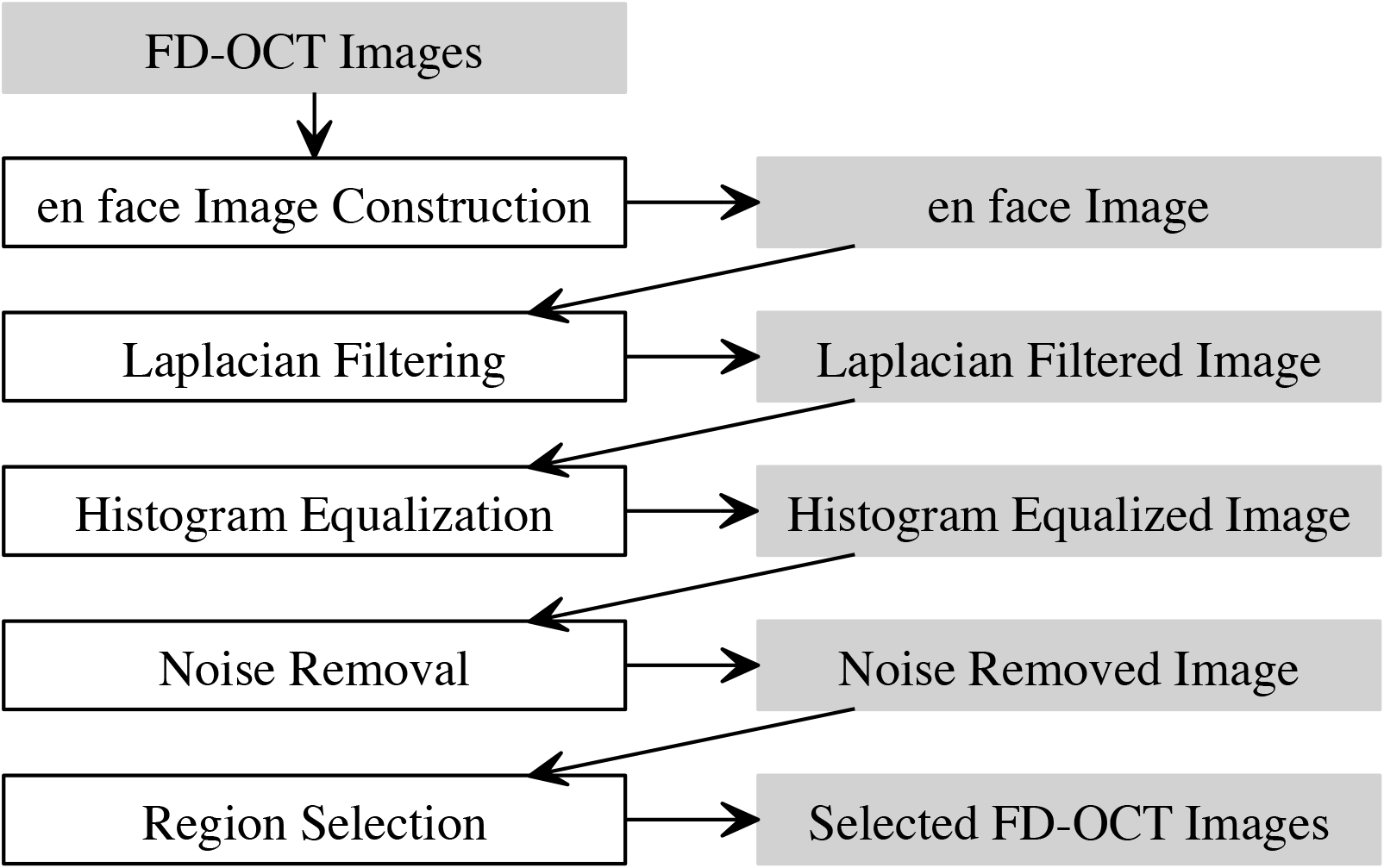

Supplement: S2 Fig — Boxes denote submodules and shades denote data. (TIF) [file pone.0124192.s002.tif]

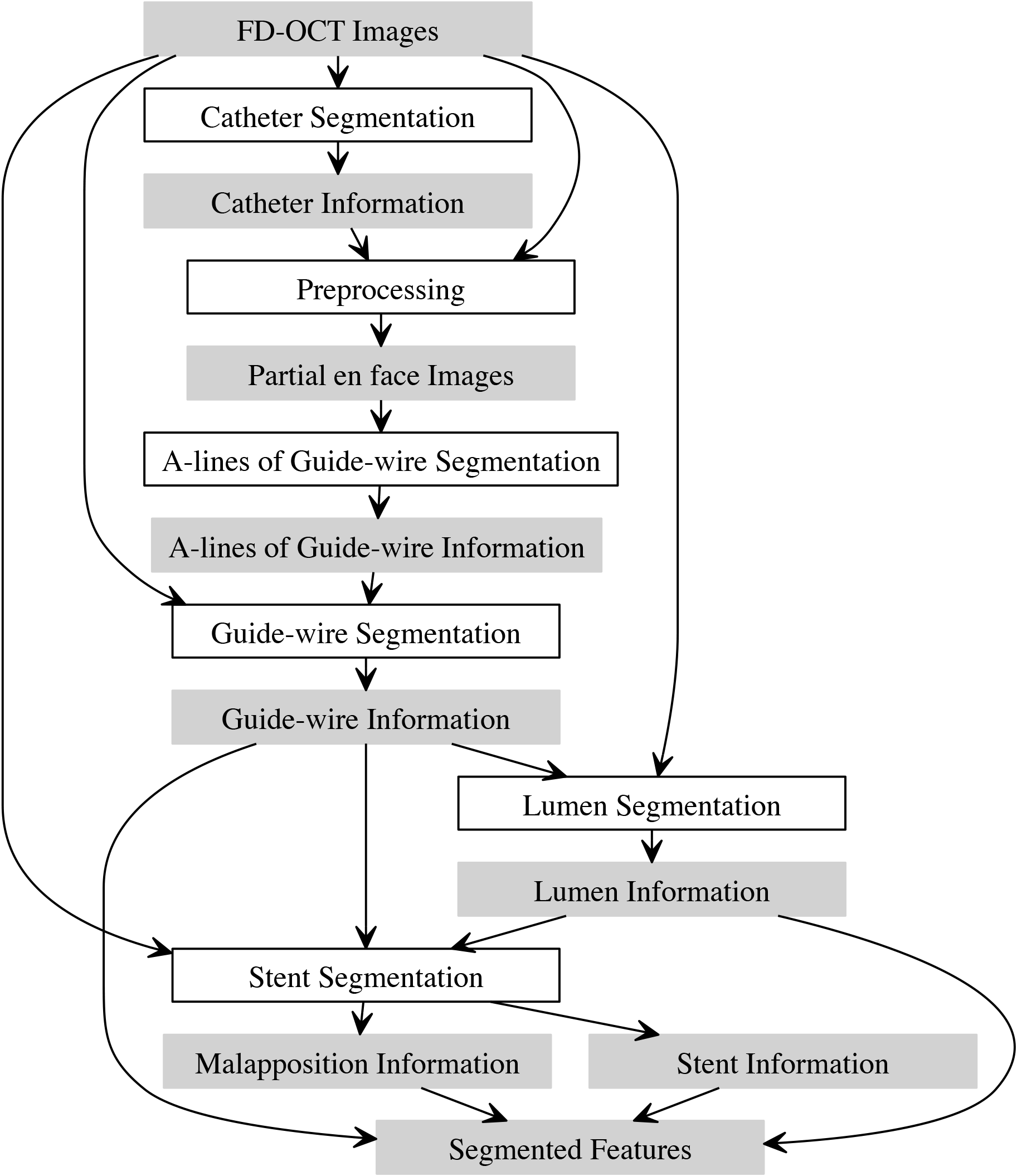

Supplement: S3 Fig — Boxes denote submodules and shades denote data. (TIF) [file pone.0124192.s003.tif]

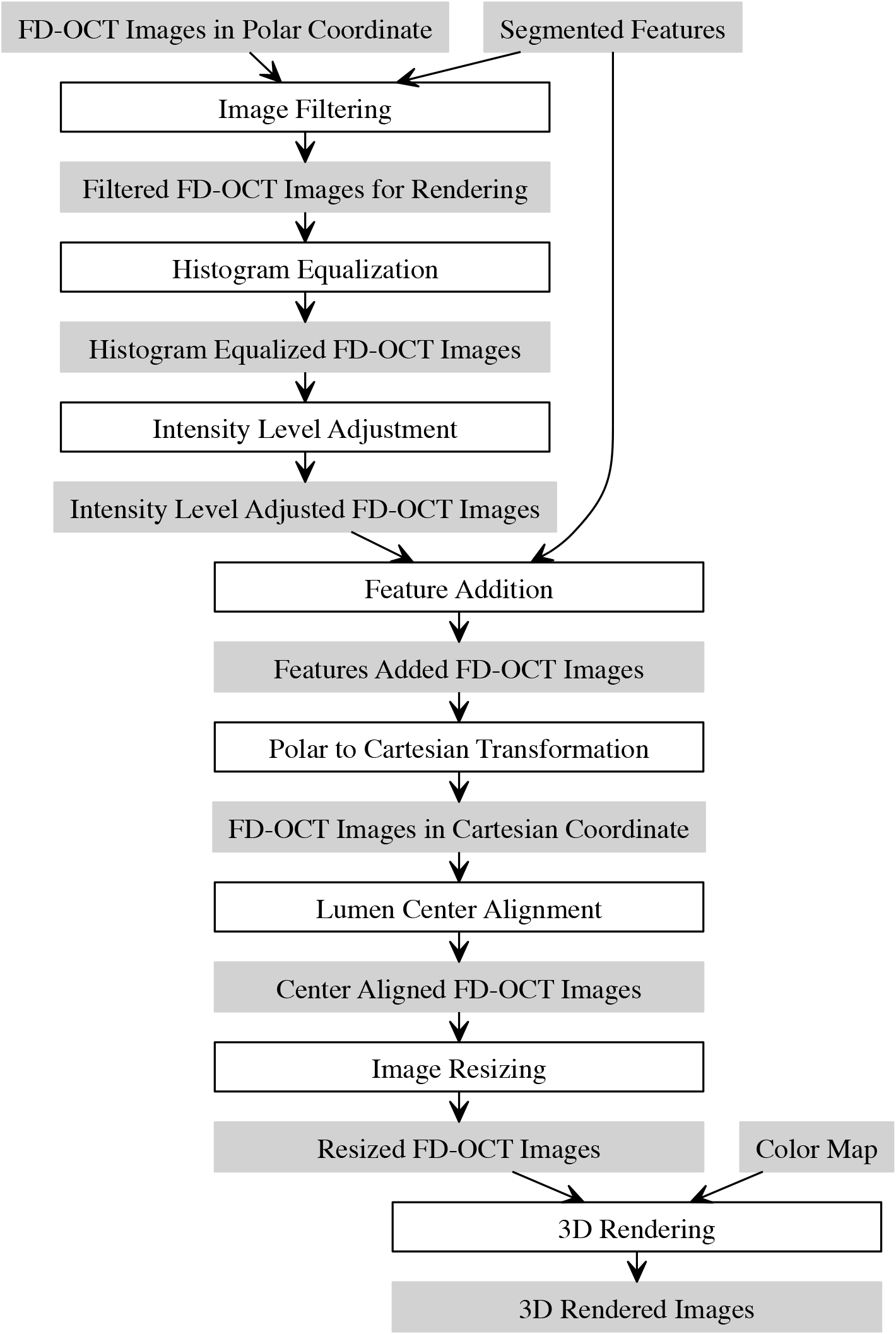

Supplement: S4 Fig — Boxes denote submodules and shades denote data. (TIF) [file pone.0124192.s004.tif]
