# Supplementary material for: GPU-Accelerated Framework for Intracoronary Optical Coherence Tomography Imaging at the Push of a Button
Source: PLoS One. 2015 Apr 16;10(4):e0124192. doi: 10.1371/journal.pone.0124192 (PMC4400174; doi:10.1371/journal.pone.0124192)
Supplement: S1 Table — (DOCX) [file pone.0124192.s005.docx]

**Table S1. Execution time (in milliseconds/frame) of each function in FD-OCT image reconstruction on CPU and GPU.**

| Function | CPU | GPU |
| --- | --- | --- |
| Background subtraction and window function application | 7.1519 | **0.2500** |
| FFT | 13.5457 | **0.4797** |
| Demodulation for frequency shift and 2x zero padding | 19.7070 | **0.3299** |
| Inverse FFT | 39.9543 | **0.4608** |
| Interpolation and dispersion compensation | 21.6398 | **0.3294** |
| FFT | 10.2970 | **0.2539** |
| Intensity image on a logarithmic scale | 43.7768 | **0.5771** |
| Total | 156.0725 | **2.6808** |
