# Supplementary material for: GPU-Accelerated Framework for Intracoronary Optical Coherence Tomography Imaging at the Push of a Button
Source: PLoS One. 2015 Apr 16;10(4):e0124192. doi: 10.1371/journal.pone.0124192 (PMC4400174; doi:10.1371/journal.pone.0124192)
Supplement: S2 Table — (DOCX) [file pone.0124192.s006.docx]

**Table S2. Execution time (in milliseconds/frame) of each function in stent region selection on CPU and GPU.**

| Submodule | CPU | GPU |
| --- | --- | --- |
| *en face* Image Construction | 2.3009 | **0.0233** |
| Laplacian Filtering | **0.0022** | 0.1437 |
| Histogram Equalization | **0.0026** | 0.0064 |
| Noise Removal | 0.0012 | **0.0001** |
| Region Selection | **0.0009** | 0.0011 |
| Total | 2.3078 | **0.1746** |
