# Supplementary material for: GPU-Accelerated Framework for Intracoronary Optical Coherence Tomography Imaging at the Push of a Button
Source: PLoS One. 2015 Apr 16;10(4):e0124192. doi: 10.1371/journal.pone.0124192 (PMC4400174; doi:10.1371/journal.pone.0124192)
Supplement: S3 Table — (DOCX) [file pone.0124192.s007.docx]

**Table S3. Execution time (in milliseconds/frame) of each submodule in feature segmentation and**

**malapposition detection on CPU and GPU.**

| Submodule | CPU | GPU |
| --- | --- | --- |
| Catheter Segmentation | **0.0150** | 0.0255 |
| Preprocessing | 17.8021 | **5.3640** |
| A-lines of Guide-wire Segmentation | 1.7877 | **0.9815** |
| Guide-wire Segmentation | **1.0153** | 7.6997 |
| Lumen Segmentation | 28.2416 | **10.5726** |
| Stent Segmentation | **18.5010** | 23.8499 |
| Total | 67.3627 | **48.4932** |
