# Supplementary material for: GPU-Accelerated Framework for Intracoronary Optical Coherence Tomography Imaging at the Push of a Button
Source: PLoS One. 2015 Apr 16;10(4):e0124192. doi: 10.1371/journal.pone.0124192 (PMC4400174; doi:10.1371/journal.pone.0124192)
Supplement: S4 Table — (DOCX) [file pone.0124192.s008.docx]

**Table S4. Execution time (in milliseconds/frame) of each group that may run faster on CPU in feature segmentation and malapposition detection.**

| Submodule | Group | CPU & Data Transfers | GPU |
| --- | --- | --- | --- |
| Catheter Segmentation | 1 | **0.0010** | 0.0144 |
| Preprocessing | 2 | 1.8188 | **0.0323** |
| A-lines of Guide-wire Segmentation | 3 | **0.0077** | 0.1935 |
|  | 4 | **0.0511** | 0.3907 |
| Guide-wire Segmentation | 5 | **0.0028** | 0.2915 |
|  | 6 | **0.2056** | 2.0271 |
|  | 7 | **0.5068** | 3.3952 |
| Lumen Segmentation | 8 | **0.0463** | 0.1305 |
|  | 9 | 16.1050 | **8.1405** |
|  | 10 | **0.1759** | 0.2005 |
| Stent Segmentation | 11 | **1.1021** | 2.4155 |
|  | 12 | 2.7781 | **0.4643** |
|  | 13 | **0.2319** | 6.6020 |
| Total |  | **23.0331** | 24.2980 |
