# Supplementary material for: GPU-Accelerated Framework for Intracoronary Optical Coherence Tomography Imaging at the Push of a Button
Source: PLoS One. 2015 Apr 16;10(4):e0124192. doi: 10.1371/journal.pone.0124192 (PMC4400174; doi:10.1371/journal.pone.0124192)
Supplement: S5 Table — (DOCX) [file pone.0124192.s009.docx]

**Table S5. Execution time (in milliseconds/frame) of each submodule in 3D visualization on CPU and GPU.**

| Submodule | CPU | GPU |
| --- | --- | --- |
| Image Filtering | 22.2690 | **3.4966** |
| Histogram Equalization | 1.8328 | **1.8163** |
| Intensity Level Adjustment | 3.2585 | **0.0430** |
| Addition of Features | 2.3128 | **0.1601** |
| Polar to Cartesian Transformation | 3.6184 | **0.2213** |
| Lumen Center Alignment | 0.7519 | **0.1782** |
| Image Resizing | **0.3414** | 0.3613 |
| 3D Rendering | 5.8948 | **0.2385** |
| Total | 40.2796 | **6.5153** |
